# Anti-oxidative effects of 4-hydroxybenzyl alcohol in astrocytes confer protective effects in autocrine and paracrine manners

**DOI:** 10.1371/journal.pone.0177322

**Published:** 2017-05-10

**Authors:** Lidan Luo, Seung-Woo Kim, Hye-Kyung Lee, Il-Doo Kim, Hahnbie Lee, Ja-Kyeong Lee

**Affiliations:** 1 Department of Anatomy, Inha University School of Medicine, Inchon, Korea; 2 Medical Research Center, Inha University School of Medicine, Inchon, Korea; 3 Department of Biomedical Sciences, Inha University School of Medicine, Inchon, Korea; Massachusetts General Hospital/Harvard Medical School, UNITED STATES

## Abstract

4-Hydroxybenzyl alcohol (4-HBA) is an important phenolic constituent of *Gastrodia elata* Blume (GEB), a traditional herbal medicine used in East Asia. Many activities have been reported to underlie the beneficial effects of 4-HBA in the brain, and in particular, its anti-inflammatory, anti-oxidative, and anti-zinc-toxic effects have been implicated in the postischemic brain. Here, the authors investigated the anti-oxidative effect of 4-HBA on astrocytes and sought to identify the underlying molecular mechanisms involved. 4-HBA dose-dependently suppressed H_2_O_2_-induced astrocyte cell death. More specifically, pre-incubation of C6 cells (an astrocyte cell line) with 100 μM 4-HBA for 6 hrs increased survival when cells were treated with H_2_O_2_ (100 μM, 1 hr) from 54.2±0.7% to 85.9±1.5%. In addition, 4-HBA was found to up-regulate and activate Nrf2, and subsequently, to induce the expressions of several anti-oxidative genes, such as, HO-1, NQO1, and GCLM. Notably, HO-1 was induced by 3.4-fold in 4-HBA-treated C6 cells, and siRNA-mediated HO-1 knockdown demonstrated that Nrf2 activation and HO-1 induction were responsible for the observed cytoprotective effect of 4-HBA. ERK and Akt signaling pathways were activated by 4-HBA in C6 cells, suggesting their involvements in protective effect of 4-HBA. In addition, 4-HBA-conditioned astrocyte culture medium was found to have neuroprotective effects on primary neuronal cultures or fresh C6 cells exposed to oxidative stress, and these effects seemed to be mediated by glial cell line-derived neurotrophic factor (GDNF) and vascular endothelial growth factor (VEGF), which both accumulated in 4-HBA-treated astrocyte culture media. Thus, the 4-HBA-mediated activation of Nrf2 and induction of HO-1 in astrocytes were found to act via autocrine and paracrine mechanisms to confer protective effects. Furthermore, given the pleiotropic effects of 4-HBA with respect to its targeting of various brain cell types and functions, it would appear that 4-HBA has therapeutic potential for the prevention and amelioration of various brain diseases.

## Introduction

*Gastrodia elata* Blume (GEB) is a member of the orchidaceae family and has been used to treat general paralysis, vertigo, tetanus, and convulsive disorder, such as, epilepsy in East Asia. 4-Hydroxybenzyl alcohol (4-HBA) is a primary constituent of GEB, and has been shown to have many beneficial effects in different animal models of neurological disorders, such as, headaches, convulsive behavior, dizziness, and vertigo [1]. Furthermore, these beneficial effects of 4-HBA have been attributed to its anti-oxidative [2,3], anti-inflammatory [4], anti-apoptotic [5], anti-excitotoxic [6], and sedative [7] effects.

The protective effects of 4-HBA have been demonstrated in various animal models of stroke, for example, a middle cerebral artery occlusion (MCAO) [3,5,8] and global cerebral ischemia [9]. Of the many pathological events found to contribute to damaging processes in the postischemic brain, oxidative stress has been demonstrated to induce neuronal cell death via the formation of reactive oxygen species/reactive nitrogen species (ROS/RNS) [10,11]. The anti-oxidative effects of 4-HBA have been reported in animal models of transient [3,5,8] and global [9] ischemia, primarily in neurons. However, considering that astrocytes exert pleiotropic functions beneficial to neurons and are important producers of antioxidants in the mammalian brain, the enhancement of astrocyte function might protect neurons from ischemic injury and improve patient’s neurological outcomes.

Nuclear factor erythroid 2-related factor 2 (Nrf2) is a well-known anti-oxidative master regulator that reduces ROS/RNS levels by up-regulating anti-oxidant/detoxification genes [11,12]. Nrf2 binds to antioxidant response element (ARE) localized in the promoter regions of a battery of antioxidant and detoxifying genes, such as, hemeoxygenase 1 (HO-1) [13], NAD(P)H:quinone oxidoreductase 1 (NQO1) [14], glutathione *S*-transferases (GST) [15], and glutamate-cysteine ligase (comprised of catalytic [GCLC] and modifier [GCLM] subunits) [16,17], and thus modulates their expressions. HO-1 is the rate-limiting enzyme that catalyzes the degradation of heme to produce biliverdin, iron, and carbon monoxide [18]. HO-1 expression is up-regulated after exposure to various noxious stimuli, such as, hypoxia, proinflammatory cytokines, heavy metals, or oxygen tension perterbation [19], and the anti-oxidative effects of its enzymatic products suppress cell death. We previously found 4-HBA has a robust neuroprotective effect in the postischemic brain and that its anti-zinc-toxicity effect in neurons and astrocytes contributes to the neuroprotection afforded by 4-HBA [Submitted]. In the present study, we investigated the anti-oxidative effects of 4-HBA in astrocytes and examined the molecular mechanism responsible, particularly with respect to Nrf2 activation, HO-1 induction, and the subsequent inductions of GDNF and VEGF.

## Materials and methods

### Cell culture and H_2_O_2_ treatment

C6 astroglioma cells (Korean Cell Line Bank, Seoul, South Korea) were grown in Dulbecco’s modified Eagle’s medium (DMEM; Sigma, St. Louis, MO) supplemented with 1% penicillin, 1% streptomycin, and 5% fetal bovine serum (FBS; Thermo, Waltham, MA) at 37°C in a humidified incubator with 95% air/5% CO_2_ atmosphere. Cells (~4×10^4^) were prepared one day before H_2_O_2_ (100 μM for 1 hr) (Sigma, St. Louis, MO) treatment.

## Primary cortical neuron culture

Experiments were carried out in strict accordance with the recommendations made in the Guide for the Care and Use of Laboratory Animals published by the National Institute of Health (NIH, USA, 2013). In addition, the animal protocol used in this study was reviewed and approved beforehand by the INHA University-Institutional Animal Care and Use Committee (INHA-IACUC) with respect to ethicality (Approval Number INHA-140522-297-1). Pregnant ICR mice were purchased from Orient Bio Inc (Gyeonggi, South Korea) and housed under a 12 hr light-dark cycle with free access to food and water. Mice were sacrificed using CO_2_ and culture preparations were then started immediately. All efforts were made to minimize animal suffering and to reduce the number of animals used. Mixed cortical cells were prepared from embryonic day 15.5 (E15.5) mouse cortices and cultured as described by Kim et al. (2011) [20]. Dissociated cortical cells were plated at a density of six hemispheres per 24-well poly-D-lysine (100 μg/ml)- and laminin (100 μg/ml)-coated plate (4×10^5^ cells per well). Cultures were maintained in MEM containing 5% fetal bovine serum (FBS) and 5% horse serum without antibiotics. On day 7 in vitro (DIV7), when astrocytes had reached confluence underneath neurons, cytosine arabinofuranoside (ara-C) was added to a final concentration of 10 μM, and culture was maintained for 2 days to halt microglial growth. Glutamine and FBS were not supplemented from DIV7 and medium was changed every other day after DIV7. Cultures were used at DIV12-14.

### Nuclear and cytoplasmic extract preparation

Nuclear and cytoplasmic extracts were prepared from C6 cells (5×10^5^) using NE-PER Nuclear and Cytoplasmic Extraction Reagents (Thermo Scientific, Rockford, IL) according to the manufacturer’s instructions. Extracted proteins were stored at -80°C.

### Cell viability assays

Viabilities of H_2_O_2_-treated C6 cells were analyzed using a MTT (3-[4,5-dimethylthiaziazol-2-yl]-2,5-diphenyl tetrazolium bromide) assay. Briefly, C6 cells were treated with H_2_O_2_ (100 μM, 1 hr) with or without co- or pre-treatment with different concentrations of 4-HBA (1, 3, 6, or 9 hrs). Twenty-four hrs later, MTT (500 μg/ml, Sigma, St. Louis, MO) was added for 1 hr, DMSO (200 μl) was then added to solubilize the formazan product formed, and optical density was read at 550 nm. To assess neuronal cell death after H_2_O_2_ treatment, we used LDH (lactate dehydrogenase) assay (Roche, Mannheim, Germany) according to the manufacturer’s instructions. Primary cortical culture supernatant (50 μl) was incubated with 50 μl LDH assay reagent for 15 min and optical densities were read at 490 nm.

### PD98059 and wortmannin treatment

C6 cells were treated with PD98059 (an ERK inhibitor) (100 μM; Calbiochem, San Diego, CA) or wortmannin (an Akt inhibitor) (1 mM; Calbiochem, San Diego, CA) for 60 min and then treated with 4-HBA.

### siRNA transfection

C6 cells (4×10^4^) were seeded in 24-well culture plates and transiently transfected with HO-1 siRNA (100 nM) using lipofectamine 3000 transfection reagent (1 μl/well, Invitrogen, Carlsbad, CA), according to the manufacturer’s instructions. Rat HO-1-specific siRNA (50-AUG GCA UAA AUU CCC ACU GCC ACG G-30 and 50-CCG UGG CAG UGG GAA UUU AUG CCA U-30) and a nonspecific siRNA (50-AUG CAC GAU AUA ACC UCA CCG UCG G-30 and 50-CCG ACG GUG AGG UUA UAU CGU GCA U-30) were provided by Santa Cruz Biotechnology (Santa Cruz, CA).

### Immunocytochemistry

C6 cells (5×10^5^) were treated with 4-HBA (100 μM) for 3, 6, 9, or 12 hrs, and then fixed with 4% paraformaldehyde (PFA) for 30 min. Anti-Nrf2 antibody (1:100, Santa Cruz Biotechnology Santa Cruz, CA) was used as the primary antibody and rhodamine-labelled anti IgG (1:200, Jackson ImmunoRes, West Grove, PA) as the secondary antibody. Cells were then stained with 6-diamidino-2-phenylindole (DAPI) (1 μg/mL, Abcam, Cambridge, UK).

### Immunoblot analysis

Cells were washed with cold PBS and lysed with RIPA buffer (50 mM Tris-HCl (pH 7.4), 1% NP40, 0.25% sodium-deoxycholate, 150 mM NaCl) containing complete Mini protease inhibitor cocktail tablet (Roche diagnostics, Basel, Switzerland). Lysates were centrifuged at 12000 rpm for 15 min at 4°C, and supernatants were loaded onto 8~12% SDS-PAGE gels. The primary antibodies used were as follows: anti-Nrf2, anti-Lamin B (both 1:1000; Santa Cruz Biotechnology Santa Cruz, CA), anti-*α*-Tubulin (1:2000, Merck Millipore, Billerica, MA), anti-HO-1 (1:3000, Enzo Life Sciences, Farmingdale, NY), anti-NQO-1), anti-GCLM (both 1:3000; Abcam, Cambridge, MA), and anti-p-ERK, anti-p-AKT, anti-ERK, and anti-AKT (1:3000; Cell Signaling, Danvers, MA).

### 4-HBA conditioned media preparation

Culture media (500 μl) of C6 cells were collected after 24 hrs of incubation with 250 μM of 4-HBA and concentrated using NANOSEP 10K, a centrifugal device for concentration (Pall Life Science, Port Washington, NY). 4-HBA-conditioned media (4-HCM) were then added to neuron-enriched primary cortical cultures or C6 cells with or without 1 μg/ml of anti-GDNF (Santa Cruz Biotechnology) or anti-VEGF antibody (Santa Cruz Biotechnology).

### ELISA for VEGF and GDNF

VEGF and GDNF in 4-HCM were quantified using a rat VEGF ELISA kit (R&D Systems, Minneapolis, MN) and a GDNF ELISA kit (Biosensis, Thebarton, Australia).

### Statistical analysis

Paired comparisons were performed using the Student’s t test and multiple comparisons by one-way analysis of variance (ANOVA) followed by the Newmane-Keuls post hoc test. Results are presented as means±SEMs and statistical difference was accepted for p values < 0.05.

## Results

### Anti-oxidative effects of 4-HBA in H_2_O_2_-treated astrocytes

**Fig 1:**
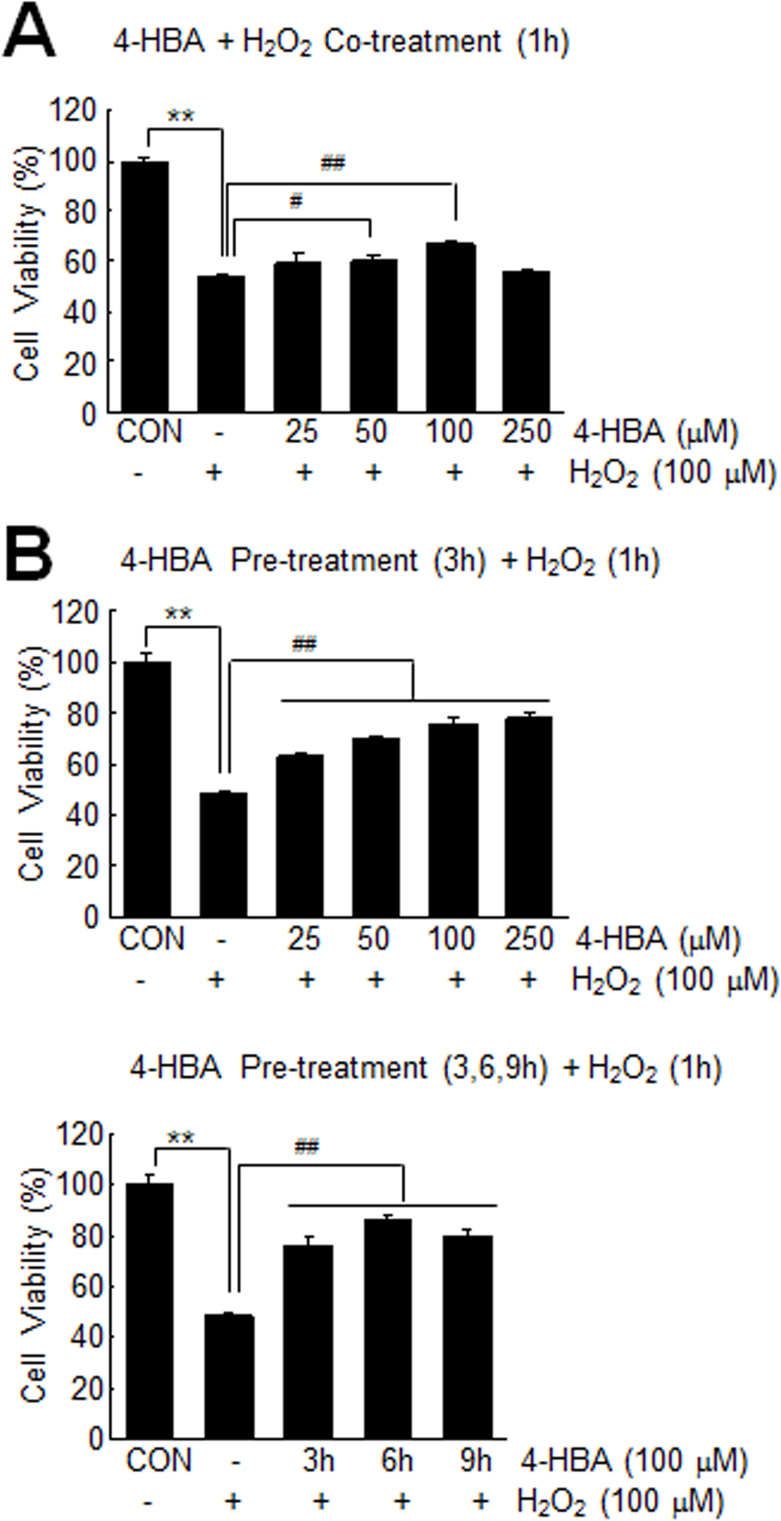
Protection of H_2_O_2_-treated C6 cells by 4-HBA. (A-B) C6 cells were treated with H_2_O_2_ (100 μM) for 1 hr in the presence or absence of 4-HBA (25, 50, 100, or 250 μM) (A), or pre-treated with 4-HBA (25, 50, 100, or 250 μM) for 3 hrs and then treated with H_2_O_2_ (100 μM) for 1 hr (B). (C) C6 cells were pre-treated with 4-HBA (100 μM) for 3, 6, or 9 hrs and then treated with H_2_O_2_ (100 μM) for 1 hr. In all experiments, MTT assays were carried out 24 hrs after H_2_O_2_ treatment. Changes in cell survival are presented as means±SEMs (n = 3). **p<0.01 versus untreated controls, ^#^p<0.05 and ^##^p<0.01 between indicated groups.

To examine anti-oxidative effect of 4-HBA in astrocytes, C6 cells were treated with H_2_O_2_ (100 μM) for 1 hr. Cell viability at 24 hrs after H_2_O_2_ treatment was reduced to 54.2±0.7% (n = 4, p<0.01) of that of treatment-naïve controls (Fig 1A). However, when C6 cells were co-treated with 4-HBA (25, 50, 100, or 250 μM) and H_2_O_2_ (100 μM) for 1 hr, the cell viabilities of 50 or 100 μM 4-HBA-treated C6 cells were slightly but significantly increased (110.4±3.3% and 122.8±2.7%, respectively, versus H_2_O_2_-treated C6 cells) (Fig 1A). Interestingly, pre-treatment of C6 cells with 4-HBA for 3 hrs prior to H_2_O_2_ treatment (100 μM, 1 hr) markedly increased cell viabilities in a 4-HBA concentration-dependent manner (Fig 1B). By pre-treating C6 cells with 100 μM of 4-HBA, cell viability increased to 157.0±4.8% of that of H_2_O_2_-treated C6 cells (Fig 1B). When cells were pretreated for 6 or 9 hrs with 100 μM of 4-HBA, cell viabilities were 177.7±3.0% and 166.1±4.1% of that of H_2_O_2_-treated C6 cells, respectively (Fig 1C). These results indicate 4-HBA confers a marked protective effect against H_2_O_2_-induced astrocyte death and that pre-treatment with 4-HBA exerts a greater effect.

#### Up-regulation and nuclear translocation of Nrf2 by 4-HBA in astrocytes

**Fig 2:**
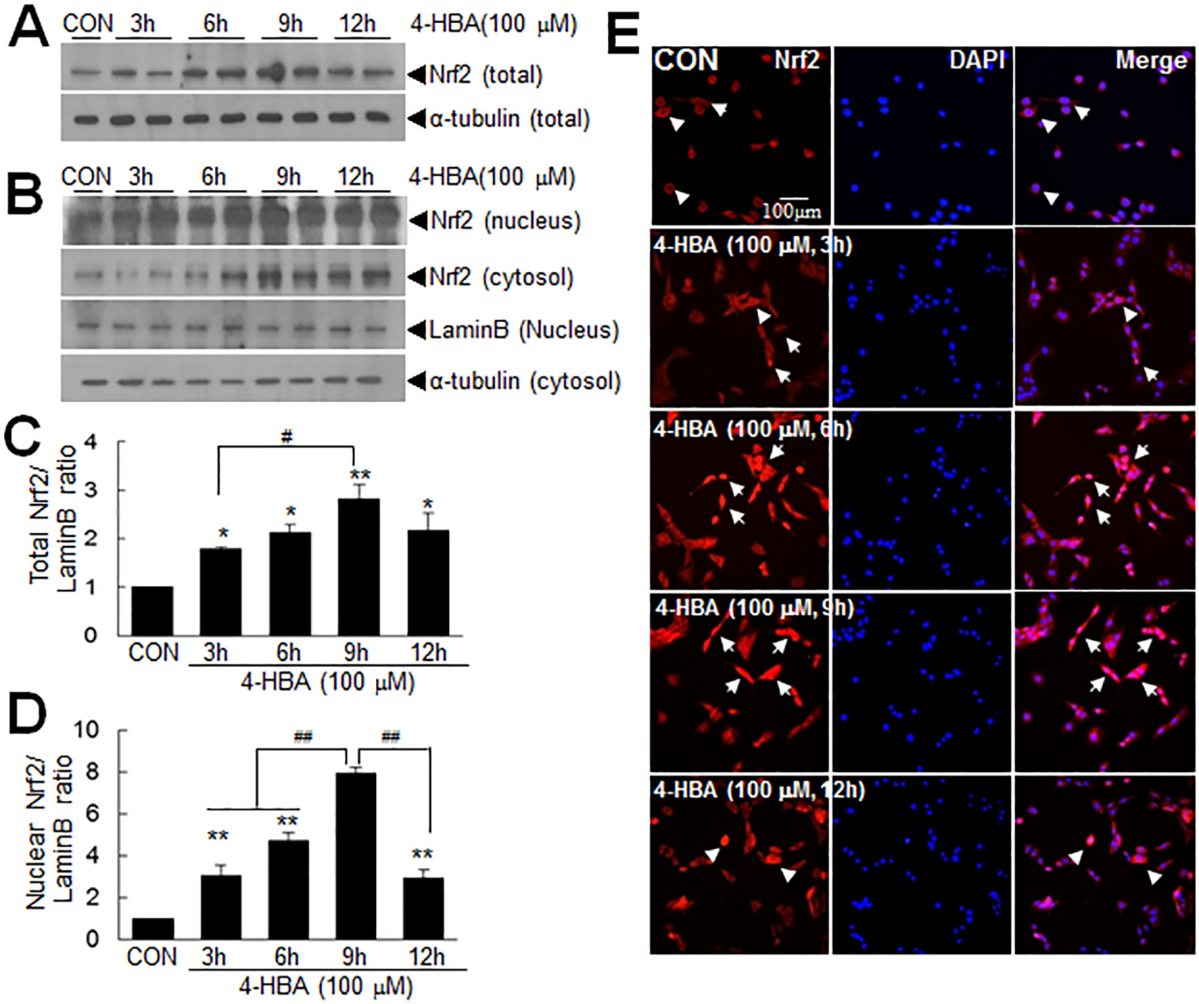
The up-regulation and nuclear translocation of Nrf2 by 4-HBA. (A, B) C6 cells were treated with 100 μM of 4-HBA for 3, 6, 9, or 12 hrs and total (A) and nuclear and cytoplasmic Nrf2 levels (B) were determined by immunoblotting. (C-D) Total (C) or nuclear (D) Nrf2 levels are presented as means±SEMs (n = 3). *p<0.05, **p<0.01 versus untreated controls, ^#^P<0.05, ^##^ p< 0.01 between indicated groups. (E) Double fluorescent staining was performed using anti-Nrf2 antibody and DAPI. Nrf2-positive cells were identified using a rhodamine-conjugated secondary antibody. Arrows indicate Nrf2 translocation from cytoplasm to nucleus and arrowheads indicate the cytoplasmic localization of Nrf2. The photographs presented are representative of three independent experiments. The scale bar represents 100 μm.

Since Nrf2 is a well-known nuclear factor that up-regulates numerous anti-oxidative genes [11,12], we examined whether 4-HBA induces the up-regulation and/or nuclear translocation of Nrf2 in C6 cells. The temporal profiles of 4-HBA-induced Nrf2 up-regulation in C6 cells, especially its nuclear levels, were examined by immunoblot analysis and immunocytochemistry after treating cells with 4-HBA. Treatment of C6 cells with 100 μM of 4-HBA for 3 hrs significantly increased total Nrf2 protein levels, and levels were further increased after treatment for 6 or 9 hrs (Fig 2A and 2C). The nuclear translocation of Nrf2 was clearly detected after 3 hrs of 4-HBA treatment (Fig 2B and 2C), and levels of nuclear Nrf2 were further increased after 6 or 9 hrs of 4-HBA treatment. These nuclear accumulation were maintained for 12 hrs (Fig 2B and 2C). Double fluorescence immunostaining with anti-Nrf2 antibody and DAPI revealed the nuclear translocation of Nrf2 after 3 hrs of 4-HBA treatment (100 μM) and Nrf2 is remained in nuclei for a further 9 hrs, but then was detected in cytoplasm (Fig 2D). These results indicate that 4-HBA induces the expression and nuclear translocation of Nrf2 in astrocytes.

### Up-regulations of genes downstream of Nrf2 in 4-HBA-treated astrocytes

**Fig 3:**
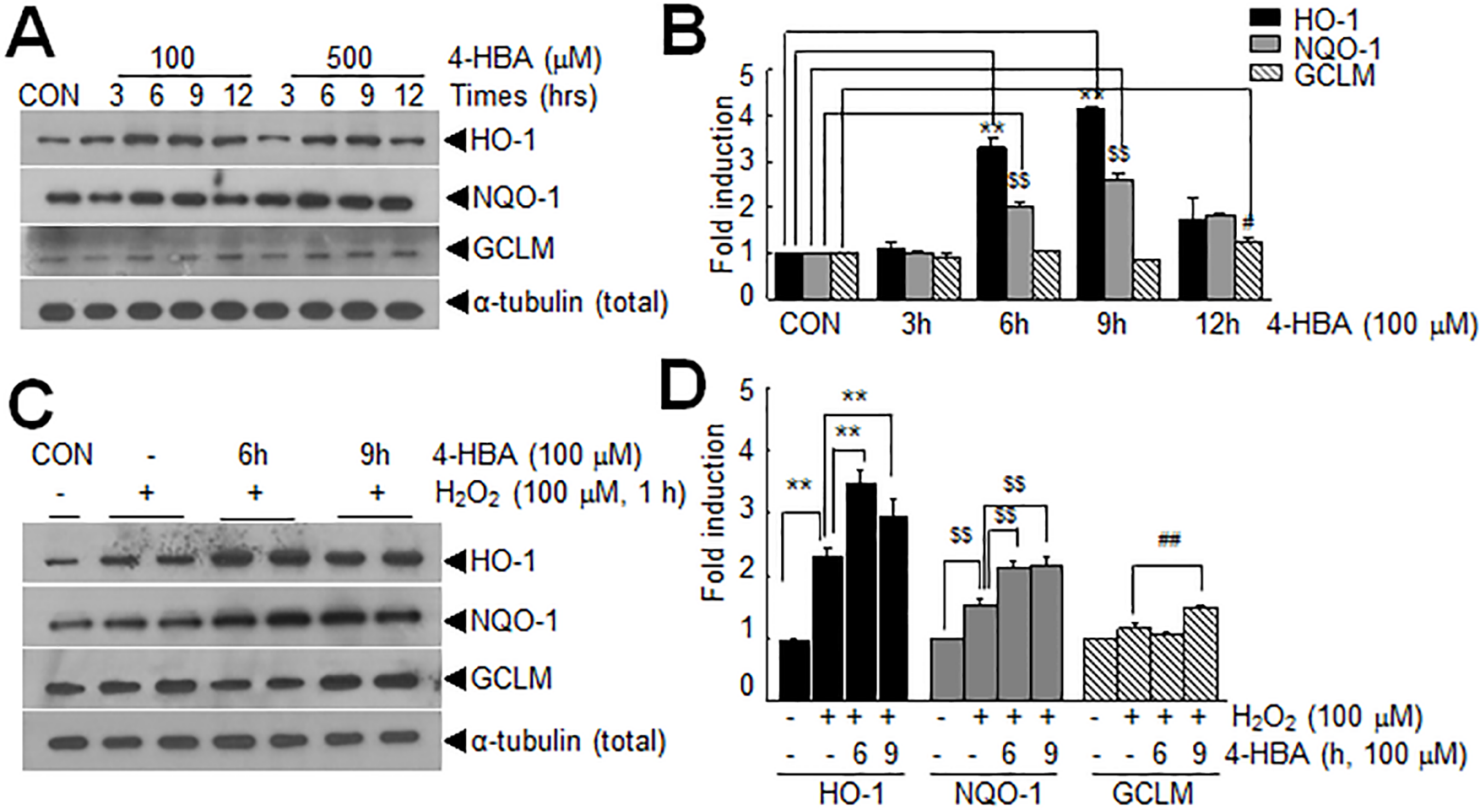
Induction of Nrf2-downstream genes by 4-HBA in C6 cells. (A-B) Cells were treated with 4-HBA (100 or 500 μM) for 3, 6, 9, or 12 hrs and protein levels of HO-1, NQO1, GCLM, and ɑ-tubulin were determined by immunoblotting. (C-D) Cells were pre-treated with 4-HBA (100 μM) for 6 or 9 hrs, treated with H_2_O_2_ (100 μM) for 1 hr, and protein levels of HO-1, NQO1, GCLM, and ɑ-tubulin were determined 1 hr later. (B, D) Protein levels determined in three independent experiments are presented as means±SEMs. **p<0.01, ^$$^<0.01, ^#^<0.05, ^##^ *p* < 0.01 between indicated groups.

The up-regulation and nuclear translocation of Nrf2 by 4-HBA prompted us to investigate whether 4-HBA induced the expression of HO-1, an important antioxidant defense enzyme modulated by Nrf2. It was found treatment with 100 or 500 μM of 4-HBA for 6 hrs significantly induced HO-1 protein in C6 cells; peak induction was detected after 9 hrs of 4-HBA treatment (Fig 3A and 3B). Similarly, protein levels of NQO1, and GCLM were also significantly increased by 4-HBA treatment (100 μM or 500 μM) with slightly different temporal profiles (Fig 3A and 3B). In H_2_O_2_-treated C6 cells, HO-1 expression was detected after 1 hr of H_2_O_2_ (100 μM) treatment (Fig 3C and 3D). Interestingly, pre-treating cells with 4-HBA (100 μM) for 6 or 9 hrs before H_2_O_2_ treatment augmented HO-1 up-regulation by H_2_O_2_ (Fig 3C and 3D). Similarly, NQO1 and GCLM protein levels were also further increased by 4-HBA pre-treatment (100 μM, 6 or 9 hrs), though to slightly different extents (Fig 3C and 3D). These results indicated that 4-HBA pre-treatment led to the rapid induction of a battery of anti-oxidative genes down-stream of Nrf2.

### 4-HBA-induced HO-1 up-regulation was responsible for the cytoprotective effect of 4-HBA in H_2_O_2_-treated C6 cells

**Fig 4:**
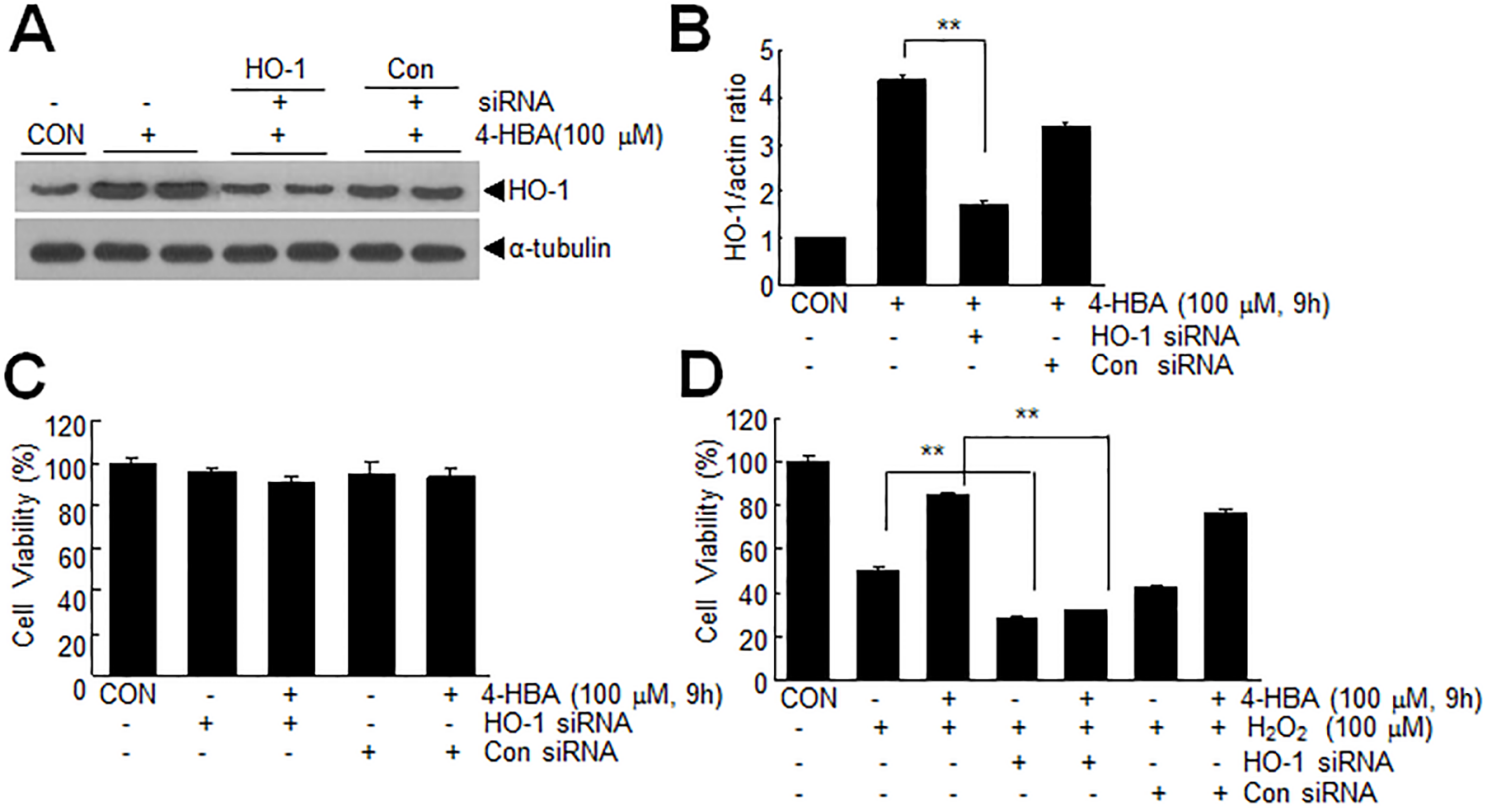
Inhibition of 4-HBA-mediated cell survival by suppressing HO-1. (A-B) C6 cells were transfected with HO-1 siRNA or control siRNA and 15 hrs later, were treated with 100 μM 4-HBA for 9 hrs. HO-1 levels were examined at 24 hrs after siRNA transfection (after 9 hrs of 4-HBA treatment) by immunoblotting. Representative photographs are presented (A) and the results obtained from three independent experiments are presented as means±SEMs (B). **p<0.01 versus siRNA-non-transfected/4-HBA treated cells. (C-D) C6 cells were transfected with HO-1 siRNA or control siRNA and 15 hrs after transfection treated with 100 μM 4-HBA for 9 hrs. Cells were then treated with serum-free media (C) or H_2_O_2_ (100 μM) (D) for 1 hr. Cell viabilities were determined by MTT assay 24 hrs after treating cells with serum-free media (C) or H_2_O_2_ (D) (48 hrs after HO-1 or control siRNA transfection). Changes in cell survivals observed in three independent experiments are presented as means±SEMs. **p<0.01 between indicated groups.

To determine whether HO-1 induction was responsible for the observed cytoprotective effects of 4-HBA in H_2_O_2_-treated C6 cells, we knocked down HO-1 with siRNA (100 nM) and 4-HBA (100 μM) was treated 15 hrs after the transfection. At 24 hrs after siRNA transfection, that is 9 hrs after treating 4-HBA, the HO-1 level was 39.1±1.7% of that in HO-1 siRNA non-transfected control cells (Fig 4A and 4B). No changes in cell viability were detected in normal cells after HO-1 siRNA or control siRNA transfection regardless of 4-HBA pre-treatment (Fig 4C). However, the increased viability observed for 4-HBA-pretreated/H_2_O_2_-treated cells was markedly suppressed by HO-1-siRNA transfection, that is, it decreased to 37.7±0.4% of that of 4-HBA-pre-treated/H_2_O_2_-treated cells (Fig 4D). Interestingly, suppressions of cell viabilities in HO-1-siRNA transfected/H_2_O_2_-treated cells with or without 4-HBA-pre-treatment were comparable (Fig 4D). In contrast, the cell viabilities of control siRNA-transfected C6 cells were no different from those of 4-HBA-pretreated C6 cells (Fig 4C and 4D). Together these results indicate that HO-1 plays a crucial role in 4-HBA-mediated cytoprotection of C6 cells treated with H_2_O_2_.

### ERK and Akt signaling pathways were involved in the 4-HBA-mediated up-regulations of Nrf2 and HO-1

**Fig 5:**
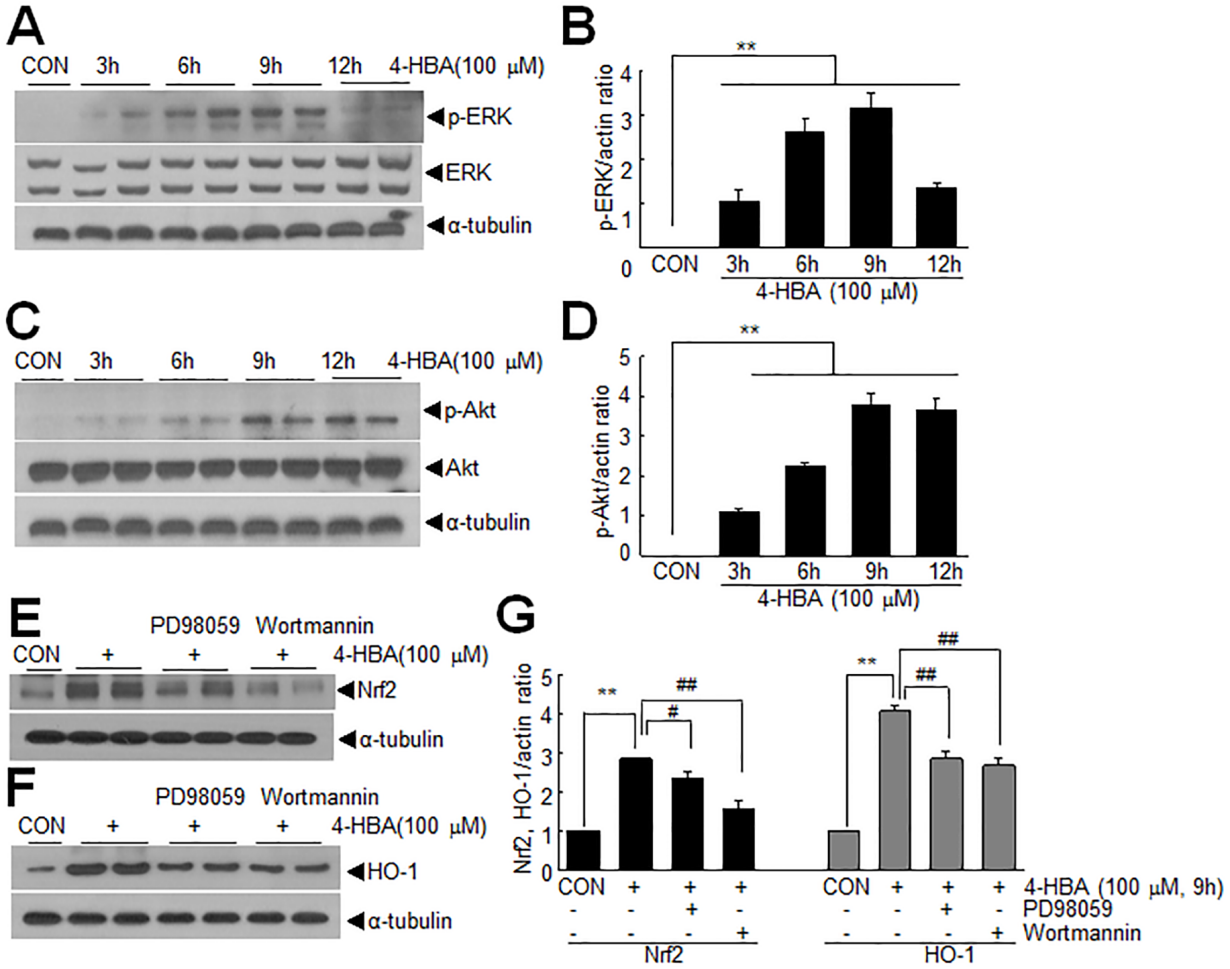
Activations of ERK and Akt during the 4-HBA-mediated inductions of HO-1 and Nrf2 in C6 cells. (A-D) C6 cells were incubated with 100 μM 4-HBA for 3, 6, 9, or 12 hrs and levels of total or phosphorylated ERK and Akt were determined by immunoblotting with anti-ERK and anti-Akt or anti-pERK and anti-pAkt antibodies, respectively. (E-G) Cells were then preincubated with PD98059 (100 μM) or wortmannin (1 mM) for 60 min, treated with 100 μM 4-HBA for 9 hrs, and total HO-1 (E, G) and nuclear Nrf2 (F, G) levels were assessed by immunoblotting. Protein levels determined in three independent experiments are presented as means±SEMs. **p<0.01 versus treatment naïve control cells, ^#^*p* <0.05, ^##^ *p* < 0.01 between indicated groups.

To identify signaling pathways mediating 4-HBA-induced HO-1 up-regulation in C6 cells, we examined the activations of various kinases known to be involved in HO-1 induction. Significant increases in phosphorylated-ERK and phosphorylated-Akt levels were observed after 3 or 6 hrs of 4-HBA treatment (100 μM), respectively, and levels were further increased until after 9 hrs of 4-HBA treatment (Fig 5A–5D), suggesting that ERK and Akt might be involved in the above-mentioned 4-HBA-mediated effects in C6 cells. Pre-treatment of C6 cells with PD98059 (100 μM) or wortmannin (1 mM) (pharmacologic inhibitors of ERK and Akt, respectively) for 60 min significantly suppressed the 4-HBA-mediated nuclear induction of Nrf2 protein (Fig 5E and 5G). In addition, the same pre-treatment also significantly inhibited 4-HBA (100 μM, 9 hrs)-induced HO-1 up-regulation (Fig 5F and 5G). These results indicate that ERK and Akt signaling pathways were involved in up-regulation/nuclear translocation of Nrf2 and subsequent induction of HO-1 in C6 cells.

### 4-HBA-mediated HO-1 induction enhanced the protein expression of GDNF and VEGF in astrocytes

**Fig 6:**
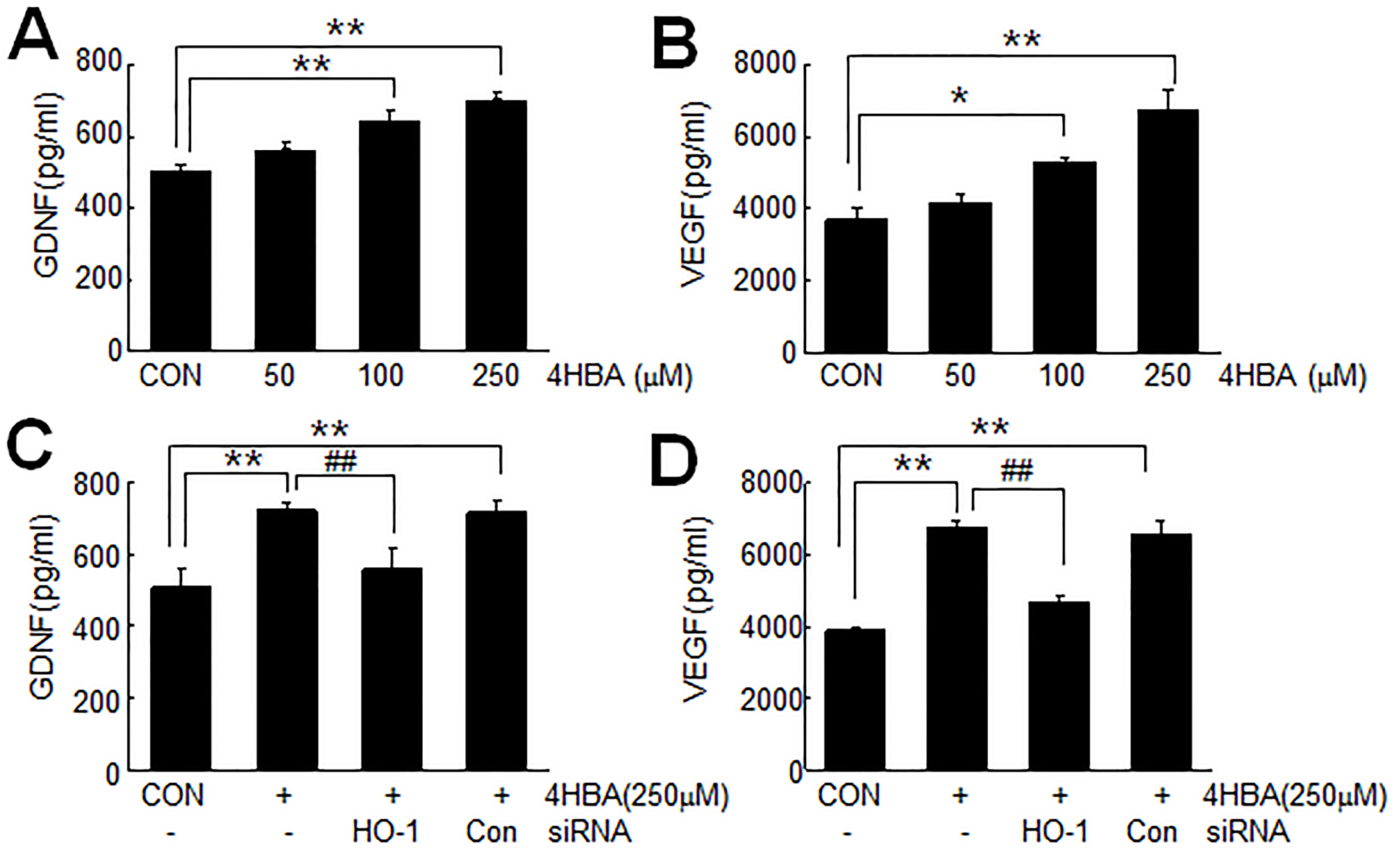
Accumulations of GDNF and VEGF in 4-HBA-conditioned astrocyte culture media. (A-B) C6 cells were incubated with 50, 100, or 250 μM of 4-HBA for 24 hrs and GDNF or VEGF protein levels in media were assayed using commercial kits. (C-D) C6 cells were transfected with HO-1 siRNA or control siRNA, and 12 hrs later, treated with 250 μM 4-HBA for 24 hrs. Secreted GDNF and VEGF protein levels in media were then measured. *p<0.05, versus the untreated control (n = 3), ^##^p<0.01 versus the 4-HBA-treated control (n = 3).

Since it has been reported that the downstream products of HO-1, i.e., bilirubin and CO, induce neurotrophic factors [21,22,23], we investigated the effect of 4-HBA-mediated HO-1 induction on the protein expressions of GDNF and VEGF in C6 cell media using protein production assay kits. Secreted GDNF protein was detected in media of C6 cells after 24 hrs of 4-HBA (100 μM) treatment and its level further increased in 250 μM 4-HBA-treated cells (Fig 6A). Similarly, dose-dependent accumulations of VEGF were also detected in 4-HBA (100 or 250 μM)-treated C6 cell media (Fig 6B). However, in HO-1 siRNA-transfected cells, 4-HBA (250 μM)-induced GDNF and VEGF protein levels in culture media were reduced to 80.0±9.3% or 68.9±5.2%, respectively, of that of non-transfected cells, indicating that 4-HBA-mediated HO-1 induction was responsible for the inductions of GDNF and VEGF (Fig 6C and 6D). In contrast, transfection of control siRNA did not suppress the inductions of GDNF or VEGF protein levels (Fig 6C and 6D). These results indicate 4-HBA-mediated HO-1 induction is responsible, at least in part, for the inductions of GDNF and VEGF in C6 cells.

### Neuroprotective effects of GDNF and VEGF in 4-HBA-conditioned astrocyte culture media

**Fig 7:**
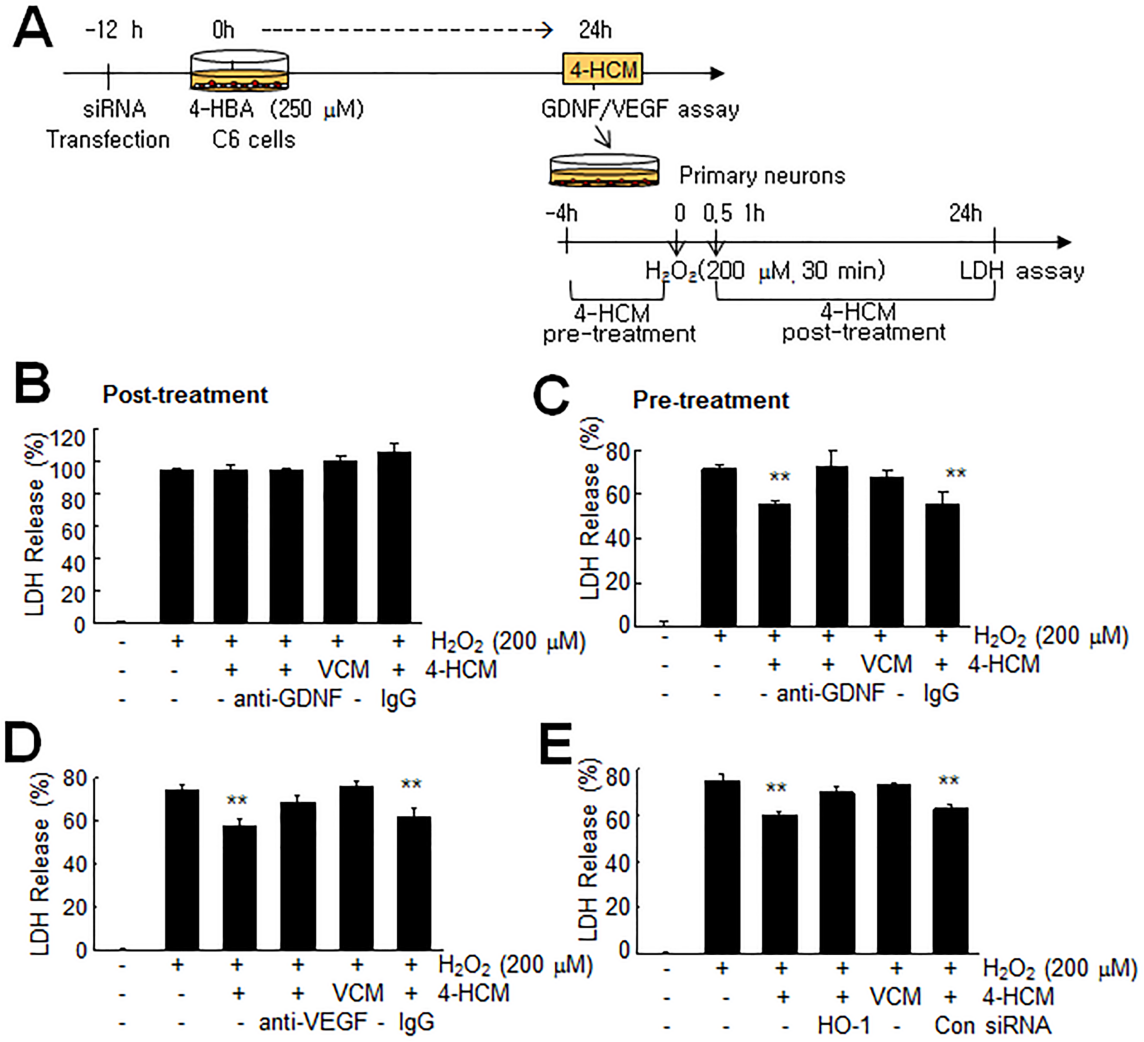
Neuroprotective effects of GDNF and VEGF in 4-HCM. (A) C6 cells were treated with 250 μM of 4-HBA for 24 hrs and 4-HCM was collected and concentrated using a NANOSEP 10K filter. Primary neuronal cultures were treated with 200 μM of H_2_O_2_ for 30 min and LDH assays were carried out 24 hrs after H_2_O_2_ treatment. (B) Primary neuronal cultures were treated with 200 μM of H_2_O_2_ for 30 min and media were then replaced with 4-HCM. LDH assays were carried out 24 hrs later. (C-E) Primary neuronal cultures were pre-treated with 4-HCM for 4 hrs and then treated with 200 μM of H_2_O_2_ for 30 min. 4-HCM was pre-incubated with 1 μg/ml of anti-GDNF (C) or anti-VEGF (D) antibody or prepared from C6 cells transfected HO-1 siRNA (E). Non-specific IgGs and non-specific siRNA were used as negative controls. LDH assays were carried out 24 hrs after H_2_O_2_ treatment. Changes in cell death are presented as means±SEMs (n = 3). **p<0.01 versus the H_2_O_2_-treated control. VCM, vehicle-conditioned medium.

Next, we examined the neuroprotective effects of GDNF and VEGF accumulation in 4-HBA-conditioned media (4-HCM). 4-HCM was collected from C6 cells treated with 4-HBA (250 μM) for 24 hrs (Fig 7A). Primary neuronal cultures were treated with H_2_O_2_ (200 μM, 30 min) with or without 4-HCM pre- or post-treatment, and neuronal cell death was examined 24 hrs after H_2_O_2_ treatment (Fig 7A). Post-treatment of primary cortical cultures with 4-HCM for 24 hrs after treating H_2_O_2_ had no protective effect (Fig 7B). However, pre-treatment of primary cortical cultures with 4-HCM for 4 hrs before H_2_O_2_ treatment suppressed H_2_O_2_-induced neuronal death to 78.1±2.9% of that of H_2_O_2_-treated control cells (Fig 7C). Importantly, these 4-HCM-mediated neuroprotective effects were not detected when the 4-HCM used was pre-incubated with anti-GDNF or anti-VEGF antibody for 4 hrs (Fig 7C and 7D) or when 4-HCM was collected from HO-1 siRNA-transfected cells (Fig 7E). In contrast, 4-HCM-mediated neuroprotective effects were detected when 4-HCM was used after pre-incubating it with control IgG or collected from non-specific siRNA-transfected cells. These results indicate that the neuroprotective effects of 4HCM were dependent on the inductions and accumulations (in media) of growth factors, such as, GDNF and VEGF, by 4-HBA.

### Autocrine protective effects of GDNF and VEGF in 4-HCM

**Fig 8:**
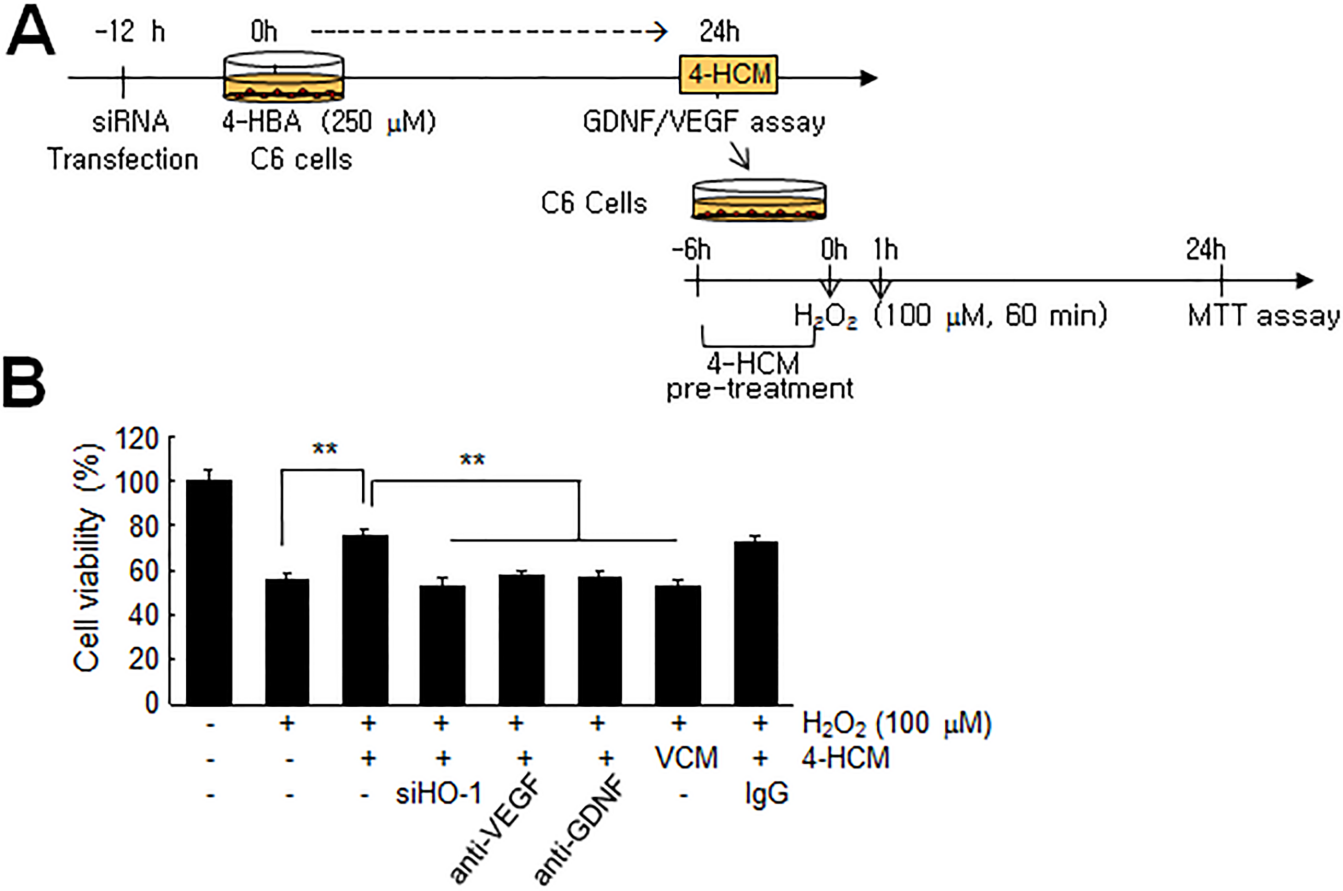
Autocrine protective effects of GDNF and VEGF in 4-HCM. (A) 4-HCM was collected as described in the legend of Fig 7A. (B) C6 cells were pre-treated with 4-HCM for 4 hrs and then treated with 100 μM of H_2_O_2_ for 60 min. 4-HCM was pre-incubated with 1 μg/ml of anti-GDNF or anti-VEGF antibody or prepared from C6 cells transfected with HO-1 siRNA before being administered to C6 cells. Non-specific IgG was used as a negative control. MTT assays were carried out 24 hrs after H_2_O_2_ treatment. Changes in cell viability are presented as means±SEMs (n = 3). **p<0.01 versus 4-HCM-pretreated/H_2_O_2_-treated cells. VCM, vehicle-conditioned media.

Next, we examined whether GDNF and VEGF accumulations in 4-HCM exert protective effects in an autocrine manner. C6 cells were treated with H_2_O_2_ (100 μM, 60 min) with or without 4-HCM pre-treatment (6 hrs), and cell viabilities were examined 24 hrs after H_2_O_2_ treatment (Fig 8A). In H_2_O_2_-treated C6 cells, mean cell viability decreased to 56.0±2.5% of that of non-treated controls, but4-HCM pre-treatment reduced this decrease to 76.2±1.9% (Fig 8B). These 4-HCM-mediated protective effects were not detected when 4-HCM was used after pre-incubating it with anti-GDNF or anti-VEGF antibody for 4 hrs or when 4-HCM was collected from HO-1 siRNA-transfected cells, but they were detected when 4-HCM was used after pre-incubating it with control IgG (Fig 8B). These results indicate that 4-HCM had an autocrine protective effect on astrocytes.

## Discussion

This study shows 4-HBA-mediated Nrf2 activation and subsequent HO-1 induction enhances the viability of H_2_O_2_-treated astrocytes and that 4-HBA-conditioned astrocyte culture media exerts a protective effect on neurons and astrocytes exposed to oxidative insults. Furthermore, GDNF and VEGF induction by HO-1 and accumulation in media were found to play important roles in this process. In addition, numerous antioxidant genes, such as, NQO-1 and GCLM, also induced by 4-HBA, might have played additional protective roles.

Excessive free radicals are known to cause cell damage in various diseases targeting the CNS. Great interest has been focused on phytochemicals found in many herbal medicinal plants that exert antioxidant activities, and 4-HBA is a good example of such a phytochemical. Indeed, several studies have shown that 4-HBA exhibits neuroprotective effects in various animal models of stroke by suppressing oxidative stress [3,8,9]. In the present study, we observed that 4-HBA had a robust anti-oxidative effect on astrocytes, wherein it up-regulated and activated Nrf2 and subsequently induced the expression of a battery of antioxidant proteins (Figs 2 and 3). Since peroxiredoxin 6 (Prdx6) and protein disulfide isomerase (PDI) were shown to be induced by 4-HBA in neurons [8,24], it would be interesting to determine whether the inductions of important anti-oxidative genes by 4-HBA are cell type specific and whether HO-1 plays a critical role in astrocytes. It is important to emphasize here that the protective autocrine and paracrine effects of 4-HCM were not obtained using HO-1 knock-downed 4-HCM (Figs 7 and 8), which indicates HO-1 played a crucial role in the 4-HBA-mediated protective effects in astrocytes.

Regarding the molecular mechanism underlying Nrf2 activation, Keap1 has been reported to modulate the ubiquitin-mediated proteosomal degradation of Nrf2 by binding Nrf2 in cytoplasm [25]. We speculate here that 4-HBA modifies the reactive cysteine residues of Keap1 (C273 and C288), which are crucial for its association with Nrf2 [26], via its electrophilic residue as has been reported for several ARE inducers, for example, t-butyl hydroquinone (tBHQ) and sulforaphane [27]. Furthermore, it is possible 4-HBA-mediated Nrf2 activation suppresses NF-kB activity by recruiting p300 to Nrf2 and depriving p300 from p65, thereby contributing to the anti-inflammatory effect of 4-HBA, as we previously showed for ethyl pyruvate and curcumin [28,29]. Further study is required to determine if this interconnection between anti-oxidative and anti-inflammatory effects is also conferred by 4-HBA.

In the present study, we observed marked Nrf2 up-regulation and activation in 4-HBA-treated C6 cells (Fig 2A and 2C). It has been previously reported that activations of ERK and PI3K/Akt contribute to the induction of Nrf2-mediated antioxidant enzyme expressions, including HO-1 [30,31]. We found that ERK activation occurred early and rather transiently, whereas Akt activation was greater and occurred later (Fig 5A–5D). In addition, Nrf2 induction was suppressed more effectively by wortmannin than by PD98059 (Fig 5E–5G), suggesting ERK and Akt act in different ways in 4-HBA-treated cells. In the case of Akt, its activation by 4-HBA and subsequent induction of Nrf2 have been previously reported in primary cortical cultures [8], which suggests this signaling pathway might be a common molecular mechanism underlying the anti-oxidative effects of 4-HBA in neurons and astrocytes. Considering the pleiotropic functions of Nrf2, including its cancer promoting effects [32,33], further studies are needed to determine specific and delicate modulations of Nrf2 inductions and activations in different contexts.

Of the various genes downstream of Nrf2, HO-1 has been reported to protect tissues by restoring redox homeostasis and reducing inflammation due to its anti-oxidant, anti-apoptotic, and anti-inflammatory effects [34,35,36]. The present study showed HO-1 induced by 4-HBA enhanced GDNF and VEGF levels in astrocytes (Fig 6), and that this process was, at least in part, responsible for both the autocrine and paracrine protective effects of 4-HCM (Figs 7 and 8). Although the actions of VEGF and GDNF seemed a little redundant, in that treatment with an antibody of either abrogated the effect of 4-HCM (Fig 7B and 7C, Fig 8B), specific functions of GDNF and VEGF have well been reported. Astrocyte-secreted GDNF protected neurons from 6-OHDA-induced cytotoxicity [37] and protected dopamine neurons from apoptosis in an animal model of Parkinson’s disease [38]. VEGF not only induced angiogenesis but modulated neuronal activity and plasticity [39] and neurogenesis in the adult brain after injury [40]. Recently, Cho et al. (2015) [41] reported 4-HBA-containing biodegradable nanoparticles improved functional blood flow in an animal model of hind-limb ischemia, and elevated levels of angiogenic inducers, such as, VEGF, HO-1, Akt/AMPK/eNOS. Therefore, we speculate the 4-HBA-mediated induction of HO-1 and subsequent releases of GDNF and VEGF might confer anti-apoptotic and pro-angiogenic effects, respectively, and thus contribute to the robust protective effect of 4-HBA observed in the postischemic brain (Luo et al., Submitted).

In addition to Nrf2 activation, direct free radical scavenging [9,42] might contribute to the anti-oxidative effect of 4-HBA. The hydroxyl groups and conjugated ring structures of 4-HBA supports the notion that 4-HBA acts as a ROS scavenger. In our previous study on Zn^2+^-induced oxidative stress, 4-HBA significantly suppressed p67 (a NADPH oxidase subunit) up-regulation and Zn^2+^-induced ROS generation in astrocytes and neurons (Luo et al., Submitted), which indicated suppression of p67 plays a role in anti-oxidative effect of 4-HBA. Additional studies are required to identify the molecular mechanisms responsible for anti-oxidative effect of 4-HBA in different cell types.

Taken together, our results provide evidence that 4-HBA protects astrocytes against oxidative stress by activating Nrf2 and subsequently inducing anti-oxidative genes, such as, HO-1, NQO1, and GCLM. Furthermore, the 4-HBA-mediated induction of HO-1 in astrocytes increased the protein expressions of GDNF and VEGF, and thus, protected neurons from excitotoxic and oxidative stress. Therefore, our findings suggest that pharmacological priming of the Nrf2-ARE pathway by 4-HBA presents a potential therapeutic means of preventing oxidative damage.
